# A thorium-229 optical nuclear clock with feedback loop

**DOI:** 10.1038/s41586-026-11084-4

**Published:** 2026-10-07

**Authors:** L. Toscani De Col, T. Riebner, I. Morawetz, F. Schneider, N. Sempelmann, J. Schlachet-Lépinay, F. Schaden, M. Bartokos, G. A. Kazakov, K. Beeks, B. Gerstenecker, M. Pimon, S. Lahs, A. Hellerschmied, T. Lercher, H. Denker, J. Premper, A. Niessner, M. Matus, M. Čížek, O. Číp, V. Lal, G. Zitzer, V. Petrov, J. Tiedau, M. V. Okhapkin, E. Peik, T. Schumm

**Affiliations:** 1 Vienna Center for Quantum Science and Technology, Atominstitut, TU Wien, Vienna, Austria; 2 Bundesamt für Eich- und Vermessungswesen (BEV), Vienna, Austria; 3 Wolfgang Pauli Institut, Vienna, Austria; 4 Institut für Erdmessung, Leibniz Universität Hannover, Hanover, Germany; 5 Institute of Scientific Instruments, CAS v. v. i., Brno, Czech Republic; 6 Physikalisch-Technische Bundesanstalt (PTB), Braunschweig, Germany; 7 Max-Born-Institute for Nonlinear Optics and Ultrafast Spectroscopy, Berlin, Germany

**Keywords:** Optics and photonics, Quantum physics, Nuclear physics, Optical spectroscopy

## Abstract

The laser-accessible nuclear transition in the thorium-229 isotope has been identified as a candidate for realizing an optical nuclear clock^1^ that might outperform current optical clocks based on electron-shell transitions in atoms or ions^2^. It is expected to be more robust against external perturbations^3,4^ and to provide enhanced sensitivity in clock-based tests of the fundamental principles of physics^5,6^. Here we realize a thorium-229 nuclear clock by stabilizing a continuous-wave laser to the 148-nm nuclear transition with rapid feedback based on absorption spectroscopy^7^. The thorium-229 nuclei are embedded in a millimetre-sized, room-temperature calcium fluoride crystal. A subharmonic of the 148-nm radiation is continuously compared with a Yb^+^ single-ion clock. The nuclear clock shows a shot-noise-limited fractional frequency instability of $3\times 1{0}^{-12}/\sqrt{\tau /{\rm{s}}}$ where *τ* is the averaging time, approaching 10^−15^ instabilities over 1 day of operation. Improvements to the instability by several orders of magnitude are projected for future devices. We use the nuclear clock to constrain models of ultralight dark matter by searching for periodic fluctuations and slow drifts in the nuclear transition energy, on timescales between 20 s and 1 day. Benefitting from the enhanced sensitivity of the thorium-229 transition, these constraints compete with the best atomic clocks concerning dark matter coupling to photons and go beyond previous measurements regarding coupling to the strong force.

## Abstract (web summary)

A thorium-229 nuclear clock is realized by stabilizing a continuous-wave laser to the 148-nm nuclear excitation with a rapid feedback loop based on absorption spectroscopy.

## Main

Over the last 70 years, atomic clocks based on microwave and optical electronic transitions have successfully served as frequency standards based on their high levels of stability and accuracy^2,8–10^. To elevate these atomic timekeeping devices to the next generation, in 2003, Peik and Tamm^1^ proposed a nuclear clock based on the extraordinarily low-energy isomeric state of the thorium-229 (^229^Th) isotope at 8.4 eV. With a lifetime approaching 1 h for a bare nucleus in vacuum, this transition offers a potential resonance quality factor of the order of 10^19^ (ref. ^11^). Additionally, the nucleus couples only weakly to perturbative fields^12^, which allows the construction of an optical clock implemented in a solid material at room temperature^13^. By doping microgram amounts of ^229^Th into a crystal host, an experimentally simple and robust clock can be created, offering a large gain in signal-to-noise ratio (SNR) compared with approaches based on a single or a few isolated nuclei, while only moderately degrading the resonance quality factor^1,4,11,14^.

A clock based on the ^229^Th nuclear transition is of great interest for testing theories beyond the Standard Model of particle physics that indicate that the fundamental constants vary over time^5^. The low energy of the ^229^Th isomer transition originates from a coincidental near-cancellation of the mega-electronvolt-scale Coulomb and nuclear contributions to the binding energies of the ^229^Th ground and excited states, making the transition frequency highly sensitive to fluctuations in the coupling constants of the interactions involved^5,6,15^. As a result, a solid-state nuclear clock in its development phase would already be competitive with or may even surpass the sensitivity of advanced comparable atomic clocks in the search for dark matter.

The realization of a nuclear clock builds upon half a century of scientific discoveries. See the reviews in refs. ^12,16^. In a research stream initiated by Kroger and Reich in 1976 (ref. ^17^), the energy of the ^229^Th nuclear transition was narrowed down from an originally conjectured value of below 100 eV to −1(4) eV (ref. ^18^), 3.5(1.0) eV (ref. ^19^) and then 7.8(5) eV (refs. ^20,21^) using gamma spectroscopy. Using the internal conversion decay channel, the existence of the ^229^Th isomer was proven in 2016 (ref. ^22^) and the energy value refined to 8.28(17) eV (ref. ^23^). An optical detection of the ^229^Th radiative nuclear decay was realized in 2022, which determined the isomer energy to be 8.338(24) eV (ref. ^24^) in the vacuum-ultraviolet (VUV) range. Thereafter, resonant laser excitation in Th:CaF_2_, Th:LiSrAlF_6_ crystals and ThF_4_ and ThO_2_ thin films was reported in 2024 and 2025 (refs. ^25–28^), quickly followed by the resolution of the nuclear quadrupole structure in Th:CaF_2_ (refs. ^13,29^).

The development of 148-nm continuous-wave laser systems in 2025 (refs. ^30,31^), in combination with highly doped ^229^Th:CaF_2_ crystals^32,33^, has enabled the measurement of the nuclear transition with absorption spectroscopy^7^. With this technique, it became possible to probe the ^229^Th nuclear transition continuously, without being limited by the long isomeric state lifetime of approximately 10 min. Moreover, absorption provides a higher detection efficiency, yielding three orders of magnitude more signal photons per second compared with fluorescence.

An optical clock is based on an atomic transition with a narrow linewidth, which is probed by a clock laser with a long coherence time. By stabilizing this laser to the atomic resonance, it can inherit the desired stability and reproducibility from the atom. Aiming for millihertz resolution and accuracy, the feedback times in this process typically extend to several seconds, requiring a pre-stabilization of the laser for higher-frequency noise to an optical cavity that has a suitable short-term stability. With the combination of these two control loops, very stable laser frequencies can be generated and then converted into a time signal through a frequency comb acting as an optical clockwork^34^.

Here, we transfer this concept from an atomic to a nuclear reference transition. Although previous studies^7,13,14^ demonstrated that it is possible to excite the ^229^Th nucleus using VUV lasers stabilized to external frequency standards, it has not yet been possible to construct a system in which the laser interrogating the ^229^Th nuclei is steered by the nuclear transition itself. Our system is a nuclear clock that operates as a stand-alone device.

## Experimental set-up

**Fig. 1:**
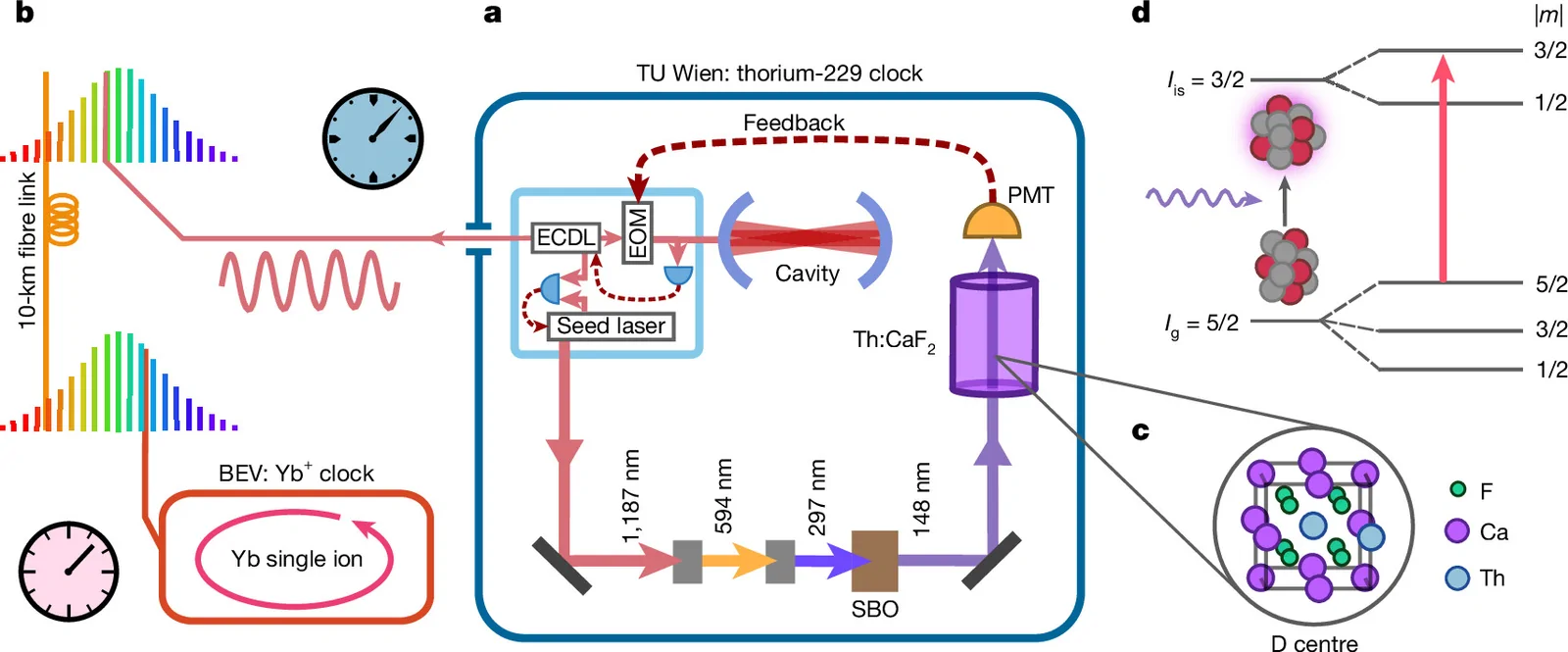
Schematic representation of the set-up. **a**, Thorium-229 nuclear clock at TU Wien. It comprises an external-cavity diode laser (ECDL), which serves as a clock laser stabilized to a high-finesse cavity through the sideband of an electro-optical modulator (EOM), and a seed laser, which is frequency-quadrupled, followed by the last SBO-based VUV frequency generation doubling. The seed laser is modulated through the offset lock to generate the error signal from the absorption of the thorium-229 nuclei in the Th:CaF_2_ crystal from the read-out of the PMT. The error signal is used as feedback to the electro-optical modulator to compensate for the long-term drift of the cavity, thus closing the nuclear clock feedback loop. **b**, The frequency is compared with that of the Yb^+^ single-ion clock at BEV by recording the beat signal on a frequency comb, which is stabilized by another high-finesse cavity (not shown) and referenced through a Doppler-compensated fibre link and a second comb to the Yb^+^ clock laser. **c**, Schematic representation of Th:CaF_2_ with a presumed thorium dimer configuration at the D centre. **d**, The ^229^Th quadrupole structure level diagram of the D centre, highlighting the investigated clock transition $| {I}_{{\rm{g}}},\,m=\pm 5/2\rangle \to | {I}_{{\rm{is}}},\,m=\pm 3/2\rangle$. *I*_g_ and *I*_is_ are the nuclear spin for nuclear ground state and isomer state, respectively.

The nuclear clock is composed of a cavity-stabilized laser for short-term stability (referred to as the clock laser) and a ^229^Th interrogation system providing long-term stability (Fig. 1a). We use a continuous-wave VUV laser source to interrogate the ^229^Th nuclei^7,30^. This laser source comprises a 1,187-nm frequency-quadrupled commercial laser (the seed laser in Fig. 1) and a single-pass second-harmonic-generation stage using a randomly quasi-phase-matched strontium tetraborate (SBO) crystal. The frequency and linewidth of the 1,187-nm interrogation laser are determined by an offset phase lock to the clock laser. We modulate the offset lock frequency to perform absorption measurements. For the clock laser, we use a high-finesse cavity-stabilized external-cavity diode laser operating at 1,187 nm. The stabilization of the clock laser to the cavity uses a Pound–Drever–Hall scheme on a sideband generated by an electro-optical modulator, allowing us to shift the frequency of the clock laser independently of the cavity resonances.

The 148-nm laser enters a vacuum chamber containing the Th:CaF_2_ crystal and the detector. We use a segment of the X2 crystal; other pieces of the same ingot were used in refs. ^13,14,25,29,35–39^. The temperature of the crystal is monitored throughout all measurements; without active stabilization it remains within an interval of plus or minus 0.5 K around 294.7 K. The detection is based on a photomultiplier tube (PMT) with a CsI photocathode, operating in photon-counting mode, placed behind the crystal for the absorption spectroscopy of the nuclear transitions^7^.

The beat frequency *f*_b_ for clock comparison and diagnostics is measured between the 1,187-nm output of the clock laser and the nearest mode of a stabilized infrared-frequency comb (Fig. 1b). The repetition rate of the comb is stabilized by locking the respective nearest comb mode to a high-finesse cavity-stabilized external-cavity laser at a wavelength of 1,542 nm. The absolute frequency of the comb is calibrated with radiation from an external-cavity laser at 1,542 nm, provided through a Doppler-compensated fibre link from the Austrian Federal Office of Metrology and Surveying (Bundesamt für Eich- und Vermessungswesen or BEV), where it is referenced to a Yb^+^ single-ion clock. We use this for comparison measurements between the Yb^+^ ion clock and the ^229^Th nuclear clock. Using the beat signal between the clock laser and the comb, we characterize the clock laser cavity drift and compensate for the dominant linear component of approximately 200 mHz s^−1^.

## Clock operation

In clock operation, the clock laser is stabilized to the interrogated nuclear transition frequency with the help of a feedback loop that corrects for residual instability or drift of the cavity. To obtain the error signal of the ^229^Th absorption line, we modulate the interrogation frequency between two frequencies separated by ${f}_{{\rm{FWHM}}}/\sqrt{3}$, with *f*_FWHM_ the full-width at half-maximum (FWHM) of the measured absorption peak, and we record the difference between the signals. Scanning is then done by moving the centre frequency over the scan range. The resulting curve has a zero crossing at the absorption peak and a near-linear slope around it. For a detailed description and an example of the error signal shape obtained, see Methods.

We can neglect absorption saturation effects as we are operating in a regime where the number of ^229^Th nuclei in the excited state is much lower than the ground-state population at any time. Therefore, the fractional absorption amplitude *A* is constant in time. The ${\rm{SNR}}\,\approx \frac{A}{\sqrt{1-A}}\sqrt{\phi }$, where *ϕ* is the number of photons detected per second. With 65 pW of VUV laser power reaching the PMT, which has a detection efficiency of 10%, and 0.75% absorption on the ^229^Th 5/2 → 3/2 transition, this equation yields SNR ≈ 17 after 1 s of measurement.

In each clock cycle, we perform a measurement at the centre frequency determined in the preceding cycle for a given integration time *T*. The result is a point on the previously measured error signal function, which we then invert to get the actual frequency deviation from the zero crossing. This deviation, expressed as an infrared frequency, is then added to the current driving frequency of the electro-optical modulator in a single adjustment step to shift the laser back to the ^229^Th resonance. The actuation of each adjustment step takes about 1 s. The size of the step depends on the integration time *T* and the stability of the high-finesse cavity used in the clock laser.

If *T* is short, the noise in the detected PMT counts will result in a high uncertainty in the estimated line centre. If *T* is long, the residual drift of the cavity during the measurement also increases this uncertainty. We can choose *T* such that the SNR of the error signal is high enough to avoid degrading the short-term stability of the clock laser inherited from the cavity. For *T* = 30 min, the feedback signal of the nuclear resonance barely impacts the fractional frequency instability at 1 s, which is then comparable with the measured cavity instability of approximately 10^−15^. In contrast to fluorescence measurements, absorption spectroscopy also enables us to use feedback times much shorter than the isomer lifetime. With *T* = 20 s, the short-term stability deteriorates more strongly from the level obtained with the cavity alone, but the laser is locked to the ^229^Th frequency on a shorter timescale. This enables faster comparison of the ^229^Th and Yb^+^ transition frequencies, which extends the accessible mass range for ultralight dark matter searches.

## Clock performance

**Fig. 2:**
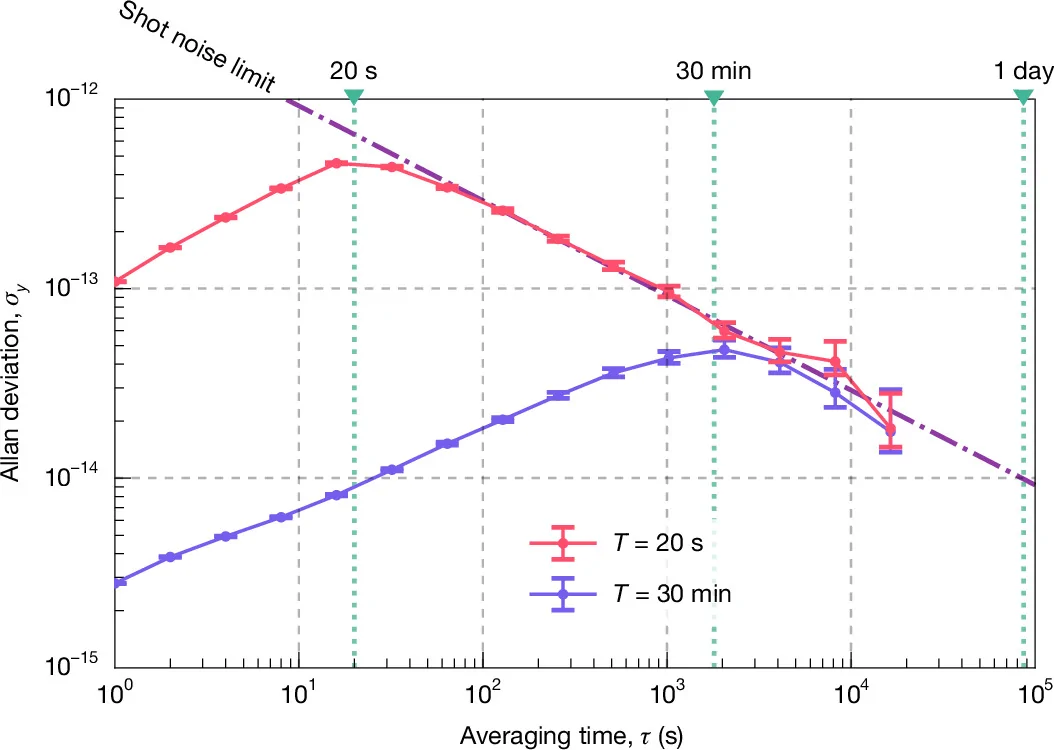
Fractional frequency instability. The fractional frequency instability for different averaging times *τ* is shown for two clock operating modes. One mode stabilizes to the 5/2 → 3/2 transition with *T* = 20 s between adjustment steps (red curve), the other with *T* = 30 min (blue curve). For *τ* > *T*, both curves follow the calculated shot-noise trend of the 5/2 → 3/2 interrogation.

The standard metric for characterizing the stability of a clock is the Allan deviation, the square root of the two-sample variance of consecutive measurements of the normalized clock output frequency. In Fig. 2 we show the overlapping Allan deviation *σ*_*y*_ (ref. ^40^) plotted versus the averaging time *τ*. For *τ* > *T*, the instability is determined by the shot-noise-limited interrogation of the ^229^Th nuclear transition ${\sigma }_{y}(\tau )\approx (\varGamma /{f}_{0})(1/\mathrm{SNR}){(\tau /{\rm{s}})}^{-1/2}$. Here *f*_0_ = 2.0204 × 10^15^ Hz is the nuclear transition frequency^7^ and *Γ* is the measured linewidth of approximately 100 kHz.

The current state of the power stability of the VUV laser system allows us to operate the nuclear clock without any intervention for 1 day. The clock operation is not affected by fluctuations of the crystal temperature of approximately 0.1 K, consistent with the temperature dependence of the 5/2 → 3/2 transition (1.8 kHz K^−1^ at 295 K) reported in ref. ^37^.

Although the clock reaches fractional frequency instabilities in the low 10^−14^ range within a continuous clock run, we observed that the reproducibility between runs on different days was limited to approximately 5 × 10^−13^. Before each clock operation run, it is necessary to realign the laser system. In our current set-up, this necessarily leads to a small change in the axis and location at which the VUV laser traverses the crystal. Therefore, each clock operation probes slightly different local regions of the X2 crystal, as the laser beam has a diameter of approximately 0.5 mm and the crystal has a diameter of 3.1 mm. To test whether this explains the reduced reproducibility, we used an *x*–*y* translation stage to perform measurements on four different crystal positions.

**Fig. 3:**
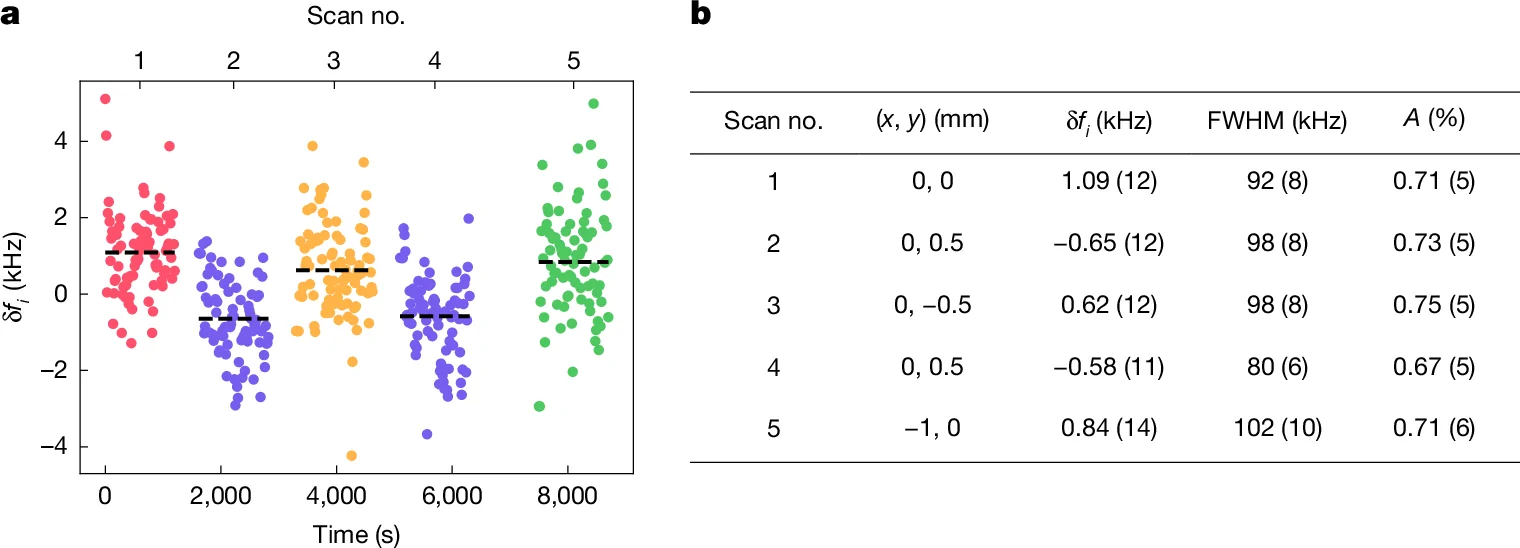
Crystal-position-dependent centre frequency. **a**, Deviations δ*f*_*i*_ of the ^229^Th line centres from the frequency average of five consecutive measurements at different crystal positions. The second and fourth datasets (blue) were obtained by operating at the same crystal position. Each point was measured for *T* = 20 s. The averages after *t*_tot_ = 20 min of data taking are the dashed black lines. **b**, The table shows the line-centre deviation δ*f*_*i*_ for each scanning position. The indicated statistical uncertainties were determined through a Monte Carlo simulation of the clock loop (Methods). The indicated FWHM of the linewidths and the fractional absorption amplitudes *A* were extracted from spectroscopy scans performed before each clock operation.

We determined the respective line centres through *T* = 20 s measurements over a total duration of *t*_tot_ = 20 min on each of the probed crystal positions. As visible in Fig. 3, the deviations δ*f*_*i*_ of the line centres from the frequency average of all five measurements differ by up to 1.7 kHz between datasets, which is well above the 0.13-kHz spread expected from statistical fluctuations in our measurement but within the uncertainty reported in previous studies of the line-centre reproducibility^14^. Remeasuring at the same crystal position (scans 2 and 4 in Fig. 3) yields a reproducible line-centre frequency. We conjecture that local strains induced by structural inhomogeneity causes these frequency shifts. In future implementations of the Th:CaF_2_-based clock, constraining the optical path through the crystal to a reproducible volume or improving doping homogeneity and minimizing strain through optimized growth conditions may enhance reproducibility.

## Constraining dark matter couplings

To address some of the most pressing open questions in fundamental physics, such as the origin and properties of dark matter, a variety of theories predict the existence of new ultralight scalar bosons^41^. Even if these bosons only have feeble interactions with the known matter particles, their influence might still be observable in high-precision experiments. For example, couplings to the electromagnetic force, strong force or quarks would lead to an apparent oscillation of the fine structure constant *α*, the quantum chromodynamics (QCD) scale parameter *Λ*_QCD_ and quark masses *m*_*q*_, respectively, with a frequency corresponding to the mass of the new boson. Because the transition energies in atoms and nuclei depend on these fundamental constants, their values would oscillate too^42^. Here the ^229^Th nuclear transition offers a big advantage: the extremely low energy of the isomer state is a result of a coincidental near-cancellation of mega-electronvolt-scale electromagnetic and strong nuclear force effects. This makes the transition very sensitive to changes in *α*, *Λ*_QCD_ and *m*_*q*_. Owing to unknown nuclear parameters, precise values of these sensitivity factors cannot at present be calculated^15,43^, but it is predicted that they will exceed those of the most sensitive atomic clock transitions by several orders of magnitude^5,6,43,44^. Thus, even if the ^229^Th nuclear clock does not yet reach the same level of frequency instability as the best atomic clocks, it already yields competitive results in searches for fundamental physics.

We searched for periodic signals in the ^229^Th transition frequency in the data from the *T* = 20 s clock operation shown in Fig. 2 (red curve). We related the variation in the infrared beat frequency *f*_b_ to the variation in the ratio between the Yb^+^ and ^229^Th transitions ${f}_{{\rm{Th}}}/{f}_{{{\rm{Yb}}}^{+}}$ through ${\partial }_{t}\,\log ({f}_{\mathrm{Th}}/{f}_{{\mathrm{Yb}}^{+}})=8({\partial }_{t}\,{f}_{{\rm{b}}})/{f}_{0}$. To search for possible oscillations of this ratio, we compute the best-fitting amplitudes ${{\mathcal{A}}}_{{\rm{f}}}=\sqrt{4{P}_{{\rm{f}}}/{N}_{\mathrm{tot}}}/{f}_{0}$ using the Lomb–Scargle method (Methods). Here *P*_f_ is the power spectrum of the beat frequencies converted into the VUV, and *N*_tot_ is the total number of statistically independent measurements. This result is sensitive to oscillations with frequencies between 1/*t*_tot_ and 1/*T*, where *t*_tot_ is the total measurement time of approximately 23 h.

**Fig. 4:**
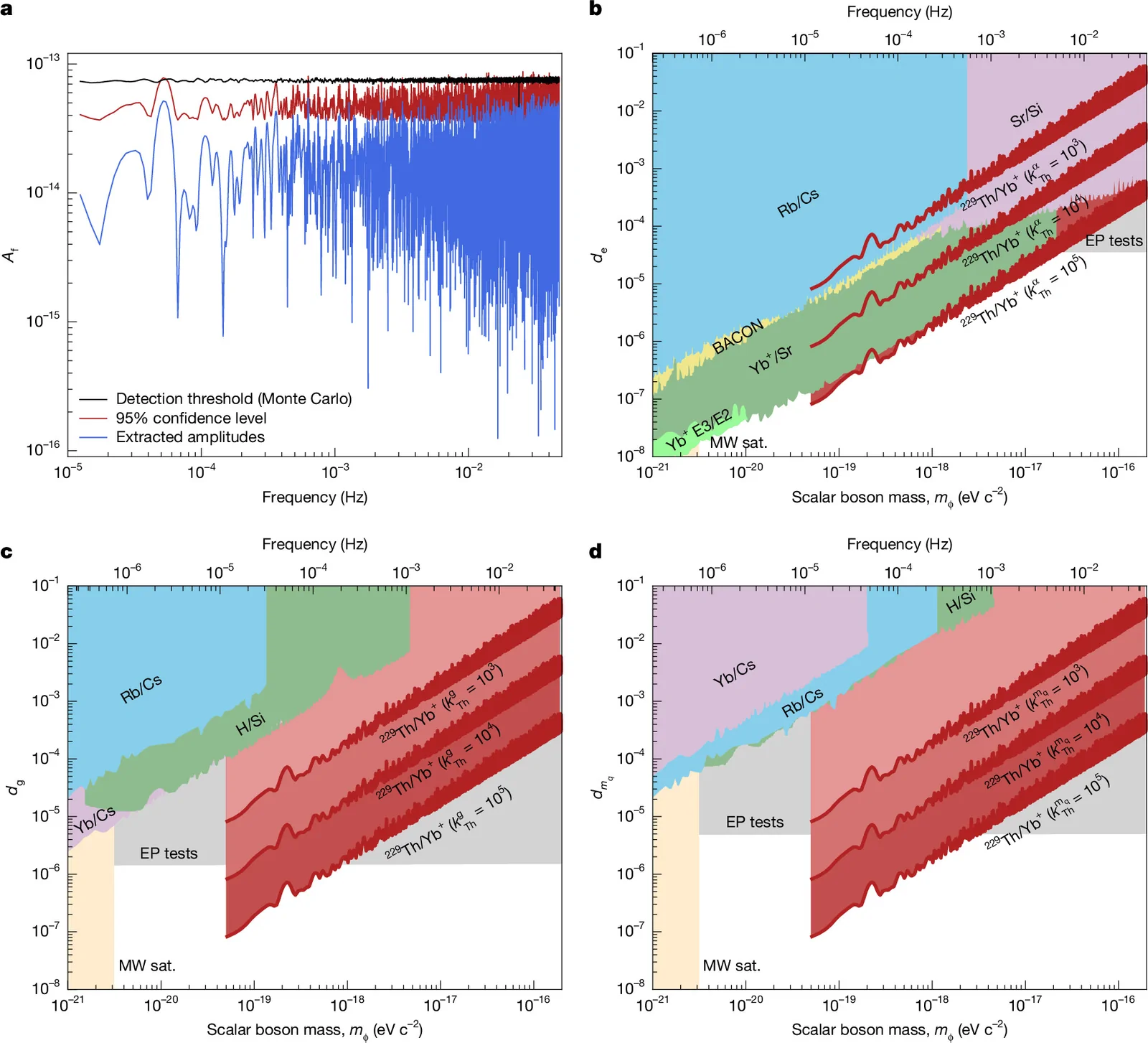
Dark matter exclusion plots. **a**, Lomb–Scargle periodogram calculated from the *T* = 20 s and *t*_tot_ ≈ 23 h measurements of the beat frequency of the ^229^Th nuclear clock and the Yb^+^ ion clock. We indicate the detection threshold that was not met at any frequency, as well as the exclusion band. We can exclude with 95% certainty that no oscillations with amplitudes above this value are present. **b**, Exclusion plot for scalar bosons with a mass *m*_*ϕ*_ and a coupling to photons with a dimensionless coupling constant *d*_e_. The shaded areas have been excluded by various clock comparisons^52–57^ (BACON: Boulder Atomic Clock Optical Network) under the assumption that the scalar boson constitutes most of the observed dark matter. Astrophysical observations of satellite galaxies of the Milky Way (MW sat.) provide a lower limit on the possible mass of such particles. Equivalence principle tests (EP)^54,58^ were able to set limits on new forces mediated by scalar particles. We followed the convention of ref. ^44^ and give several bands corresponding to sensitivity factors ${k}_{{\rm{Th}}}^{\alpha }$, ${k}_{{\rm{Th}}}^{g}$, ${k}_{{\rm{Th}}}^{{m}_{q}}$ of 10^3^, 10^4^ and 10^5^. The sensitivity factors of the Yb^+^ reference clock ${k}_{{{\rm{Yb}}}^{+}}\approx 1$ are negligible in comparison^59^. **c**, For the variation of the QCD confinement scale *Λ*_QCD_, the sensitivity of the ^229^Th transition *d*_g_ remains the same whereas limits from atomic clocks become significantly weaker. Here the ^229^Th nuclear clock is able to test a region of the parameter space that was previously not accessible. **d**, Also for the variation of the mass of the up and down quarks *m*_*q*_, the ^229^Th clock is able to provide new bounds on the coupling constant *d*_*m*__*q*_.

To identify the statistical significance of the peaks in the Lomb–Scargle periodogram, we used Monte Carlo simulations of the clock feedback loop to calculate a 5% detection threshold. As no amplitudes exceeded this detection threshold, we established an upper limit for the amplitude of oscillations in the investigated frequency range (Fig. 4a and Methods). The upper limit can further be used to restrict possible couplings to scalar dark matter. The size of the coupling constant *d*_e_ for the interaction between a scalar boson *ϕ* and photons relates to the measured power spectrum *P*_f_ as follows (Methods):

$$\begin{array}{c}{d}_{{\rm{e}}}=\frac{1}{{k}_{\mathrm{Th}}^{\alpha }\,{f}_{0}}\sqrt{\frac{{P}_{{\rm{f}}}}{{N}_{\mathrm{tot}}}}\frac{{m}_{\phi }}{1.20\times 1{0}^{-31}\,\mathrm{eV/}{c}^{2}}\\ \,=\,\frac{{{\mathcal{A}}}_{{\rm{f}}}}{{k}_{\mathrm{Th}}^{\alpha }}\frac{{m}_{\phi }}{2.40\times 1{0}^{-31}\,\mathrm{eV/}{c}^{2}}.\end{array}$$

The first factor contains the ^229^Th-specific enhancement through the large sensitivity factor ${k}_{{\rm{Th}}}^{\alpha }$ and transition frequency. The second factor indicates how the uncertainty decreases with reduced noise and higher statistics. The final factor leads to the decreasing sensitivities for higher boson masses *m*_*ϕ*_ and contains a suppression factor that is the same for any clock measurement, where *c* is the speed of light. For variations of the fine structure constant *α*, which corresponds to a scalar coupling to photons, the nuclear clock presented in this work imposes limits like those of the best comparable atomic clocks (Fig. 4). The transition frequencies of atomic clocks are less dependent on nuclear properties, even for those clocks that are based on hyperfine transitions, like the Cs clock. Thus, their sensitivities to variations of *Λ*_QCD_ and *m*_*q*_ are lower. The nuclear clock presented in this work reached a factor of 10 to 1,000 deeper into the parameter space than earlier experiments with atomic clocks. Compared with ref. ^44^, in which measurements of the ^229^Th line position and shape over 10 months were analysed, our measurement offers an improvement of around one order of magnitude in the accessible frequency range. The fast feedback of the absorption scheme allowed us to directly probe masses that could previously only be restricted through a line-shape analysis^44,45^. In future clock measurements, further enhancements in sensitivity can be expected. The frequency limits of the Lomb–Scargle periodogram are given by 1/*t*_tot_ and 1/*T*. Therefore, lower frequencies could be reached by operating the clock for longer, whereas higher frequencies are accessible by reducing the cycle time. The sensitivity over the whole frequency range scales with the square root of the number of statistically independent measurements *N*_tot_.

Apart from searches for oscillations, we analysed the frequency ratio ${f}_{{\rm{Th}}}/{f}_{{{\rm{Yb}}}^{+}}$ data for possible slow drifts. A linear fit of the *T* = 20 s and *t*_tot_ ≈ 23 h data gave a slope of (2 ± 4) × 10^−14^ d^−1^, consistent with 0. Using the same sensitivity constants introduced above, this corresponds to linear drifts of $\log \alpha$, $\log \,{\varLambda }_{\mathrm{QCD}}$ and $\log \,{m}_{q}$ of (2 ± 4)/*k*_Th_ × 10^−14^ d^−1^. The aforementioned line-centre reproducibility will be a limiting factor when extending this search to months or years.

## Prospects for future solid-state ^229^Th nuclear clocks

The performance of the ^229^Th clock could be straightforwardly improved by increasing the VUV laser power. The power limitation of the laser source used in our experiment is defined by the spontaneous domain-inversion structure of the SBO crystal. Only a fraction of the domains contributes to the frequency upconversion of the 297-nm radiation.

One order of magnitude gain in the VUV power could be achieved by using the presented randomly quasi-phase-matched SBO in an enhancement cavity, which is limited due to optical losses at 297 nm. Further enhancement of the VUV power to the hundreds of nanowatts level could be reached by using commercially available 3-W ultraviolet lasers.

Higher VUV power levels could be obtained using four-wave mixing in Cd vapour^31^, for which 300 nW has already been reported, or by fabricating patterned SBO^46^ and BaMgF_4_ crystals^47^. For a first-order periodically poled nonlinear crystal, a VUV power of at least 1 μW can be expected. However, this has not yet been demonstrated due to technical challenges in the submicrometre periodic poling.

The shot-noise-limited clock instability scales with ${\sigma }_{y}(\tau )\approx (\varGamma /{f}_{0})$ $(1/\mathrm{SNR}){(\tau /{\rm{s}})}^{-1/2}$. Beyond increasing the laser power, there are two other ways to improve the clock performance: increasing the absorption and decreasing the linewidth *Γ*. We operate in a low-saturation regime, where the fractional absorption amplitude *A* does not change with time. This will be the case as long as the number of photons that are absorbed during the lifetime of the isomeric state is much lower than the number of ^229^Th nuclei in the beam path. In the current experiment, approximately 3 × 10^8^ photons were absorbed in 10 min, although there were approximately 10^16^ ^229^Th nuclei in the beam path. Therefore, absorption measurements are expected to operate far from saturation, even if the available laser power increases significantly with further laser developments.

In ref. ^14^, it was found that *Γ* increases with the ^229^Th density *ρ*_Th_. The absorption *A* also depends on *ρ*_Th_ and increases as $A=1-\exp (-\bar{\sigma }L{\rho }_{{\rm{Th}}}\,\chi )$. Here *L* is the length of the light path through the crystal, *χ* quantifies the percentage of Th atoms that are in a D centre (for X2, 58.6%; ref. ^29^) and $\bar{\sigma }$ is the absorption cross section, which itself scales with *Γ*^−1^. These scalings imply that an optimal *ρ*_Th_ exists for which *σ*_*y*_ is minimized. The shot noise limit resulting from this theoretically optimal doping density and *χ* = 1 is only a factor of 2 lower than that of our current crystal, X2. Therefore, no large improvements in *σ*_y_ can be expected by modifying *ρ*_Th_ in future Th:CaF_2_ crystals. It should be possible with longer crystals and VUV cavities to increase the path length *L* by a factor of 10, resulting in an order of magnitude higher SNR. With this, and a laser power of 1 μW, ^229^Th-doped CaF_2_ crystals could reach a fractional frequency instability of approximately $1{0}^{-15}/\sqrt{\tau /{\rm{s}}}$.

Going beyond Th:CaF_2_, there are other possible host crystals. We expect crystals containing Th as part of the stoichiometric crystal structure, such as ThF_4_, to have reduced linewidths. In such crystals, ^229^Th is not a dopant and, thus, does not distort the crystal structure, presumably reducing the inhomogeneous broadening of the nuclear transition. This is also expected to improve the line-centre reproducibility.

Another relevant contribution to the linewidth is the magnetic interaction with neighbouring F-19 nuclei, which causes broadenings in the range 0.1–1 kHz (refs. ^4,27^). Even smaller linewidths might become accessible with spinless solids^48^. To benefit from these improvements, the linewidth of future VUV lasers needs to be narrowed down equivalently. Using a conservative linewidth estimate of *Γ* = 1 kHz, a crystal with similar doping density and dimensions as the one presently used would yield an optical depth $\bar{\sigma }L{\rho }_{{\rm{Th}}}\,\chi \approx 1$. With this, and using a 100-pW laser with a high stability cavity, a fractional frequency instability of approximately $1{0}^{-16}/\sqrt{\tau /{\rm{s}}}$ could be reached. This would bring the frequency instability of the nuclear clock to the same level as state-of-the-art optical atomic clocks^49–51^ while still maintaining the form factor and simplicity of a solid-state clock. Compared with the nuclear clock introduced in this work, this would enable a four orders of magnitude increase in sensitivity to the variation of the fundamental constants.

## Methods

### Laser set-up

In the ^229^Th interrogation system, a commercial laser (FHG-TA Pro, TOPTICA) provides an output power of approximately 500 mW at a wavelength of 296.8 nm, frequency-quadrupled in two doubling stages and one amplification stage from a diode seed laser at 1,187 nm. The final second-harmonic-generation process is based on nonlinear frequency conversion in an SBO crystal kept under a high-purity N_2_ atmosphere (purity 5.0). Fundamental radiation still present in the beam after the final second-harmonic-generation step is separated using three dielectric-coated mirrors. For the absorption measurements, we used a head-on type PMT (R6835, Hamamatsu) mounted inside the vacuum chamber and operated at 2.5 kV. We used the same segment of the X2 sample^60^ as in ref. ^7^. The segment in use has a cylindrical geometry with a diameter of 3.1(1) mm and a length of 4.2(1) mm and was oriented such that the beam traversed along the centre line of the piece. The measured average concentration of ^229^Th in the segment was 6.6(5) × 10^15^ mm^−3^.

The seed laser of the FHG-TA was locked to the clock laser (CLS, TOPTICA) through an offset frequency phase lock. A fast photodiode with a bandwidth of 10 GHz measured the beat frequency between the two lasers, which was then mixed with a reference frequency. The fast loop of this lock actuated on the seed laser diode current, and the slow loop was used to steer the grating of the laser diode. The laser was scanned by modulating the reference frequency.

Like the actuators of the offset lock, the Pound–Drever–Hall lock of the clock laser to the cavity was also configured such that the fast feedback loop actuated on the laser diode current, whereas the slow feedback signal of the Pound–Drever–Hall scheme controlled the position of the grating for optical feedback to the laser diode.

For clock comparison measurements and drift rate measurements of the cavity, the beat frequency *f*_b_ of the clock laser with the frequency comb (FC1500-250-ULN, Menlo) was recorded using a dead-time-free frequency counter (K+K FXE) operated in Π-type counting mode^61,62^ at a gate time of 1 s. Our method of comparing the thorium clock at TU Wien with the Yb^+^ clock (TOPTICLOCK, TOPTICA)^63^ at BEV follows the scheme described in ref. ^7^. The Yb^+^ clock was based on the 435.5-nm E2 transition of a single ^171^Yb ion.

All frequency synthesizers and counters in the ^229^Th clock system and the frequency comb were referenced to the 10-MHz signal of a commercial Rb clock (FS725, SRS).

To perform an absorption measurement at a specific frequency, we modulated (square wave) the offset lock frequency between the seed laser diode and the clock laser with 10 Hz between the target frequency and an off-resonance frequency. Each modulation cycle was also reflected in a synchronization signal connected to a time-resolved pulse counter (TimeTagger Ultra, Swabian Instruments). The modulation ensured that power fluctuations in the laser output did not affect the measurement. The detection set-up consists of a PMT, a radiofrequency amplifier and the pulse counter, which binned the arriving pulses from the PMT relative to the last synchronization edge. The absorption was then calculated by subtracting the on-resonance counts from the off-resonance counts and dividing by the off-resonance counts.

### Clock operation

**Extended Data Fig. 1:**
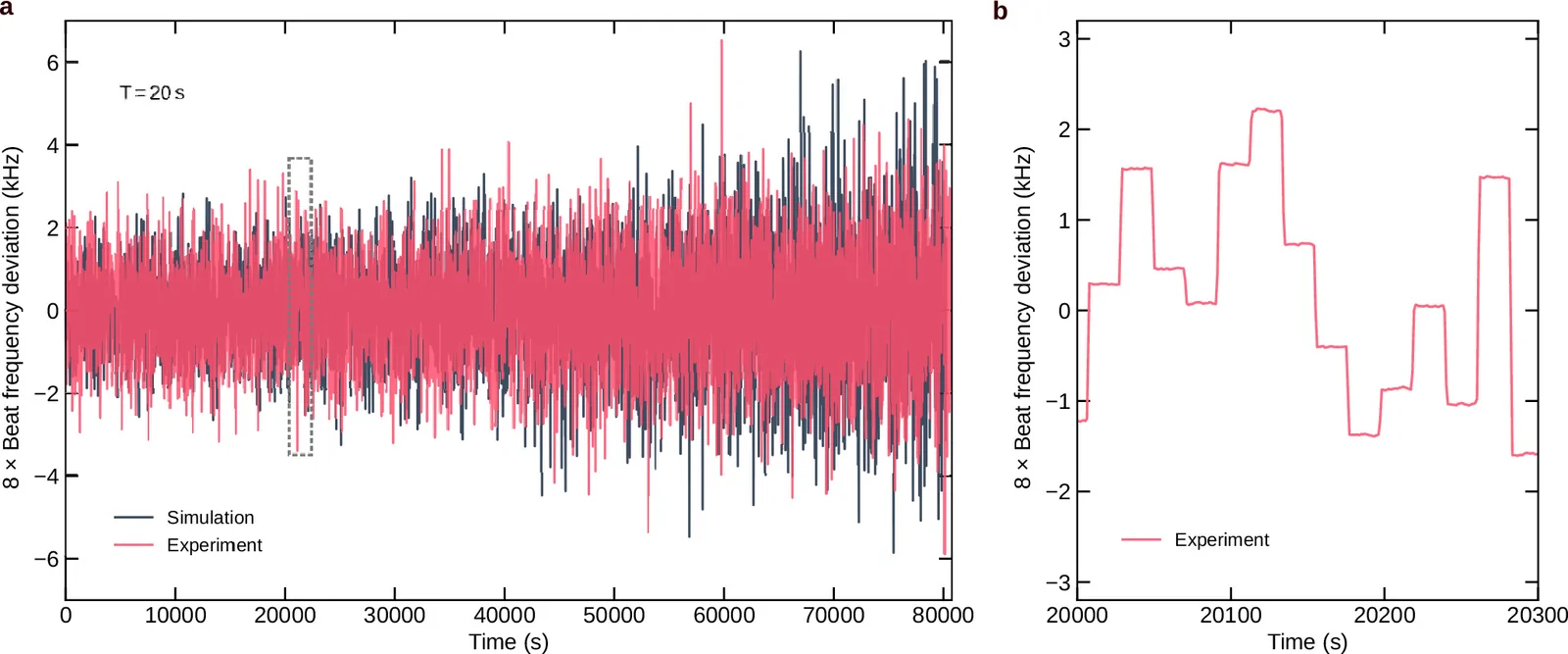
Beat frequency measurement. **a**, The deviation of the IR beat frequency from its mean value for the *T* = 20 s, 5/2 → 3/2 clock operation mode is shown (red). It is multiplied by a factor of 8 to indicate its effect on the ^229^Th line-center estimate. We additionally show one set of simulated beat frequencies (black). **b**, A zoom-in on a shorter timescale (300s) highlights the signal adjustment steps.

**Extended Data Fig. 2:**
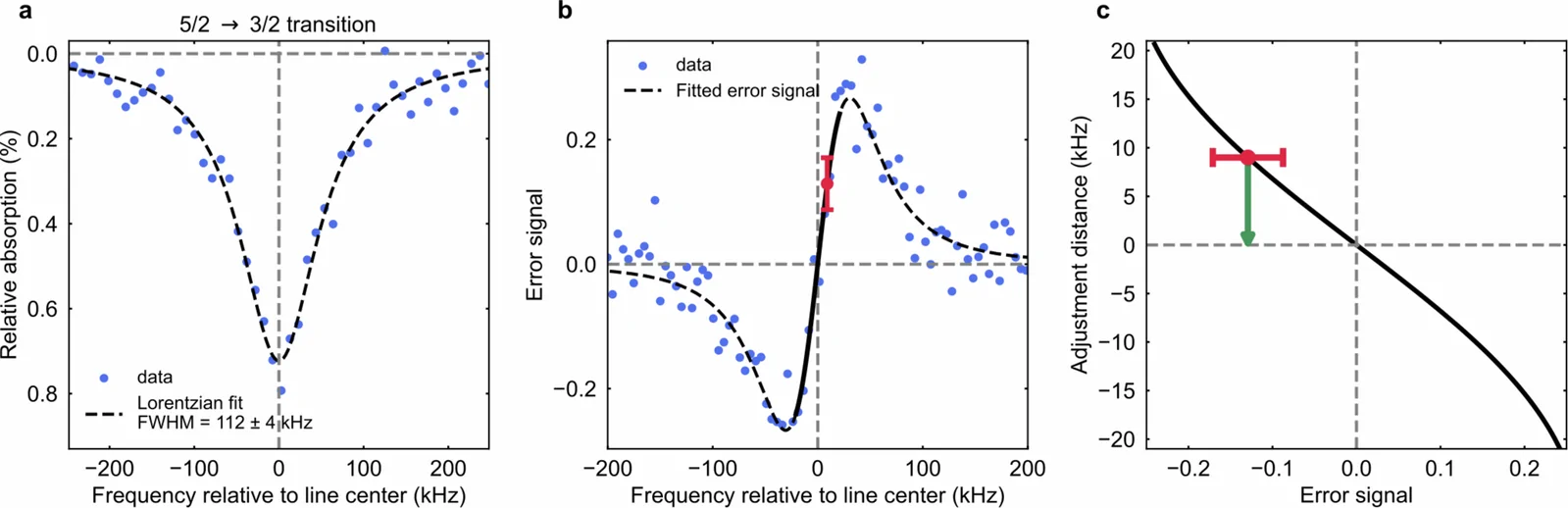
Calibration and principle of clock operation. **a**, Absorption measurement of the 5/2 → 3/2 nuclear clock transition with an integration time *T* = 3 s per data point and an off-resonance frequency-offset of +300 kHz, expressed as a function of the offset lock frequency translated into the VUV. This is used to determine the linewidth for the error signal scan. **b**, Error signal measured with an integration time *T* = 3 s per data point and a constant frequency modulation of Δ*f*_*ol*_  = 34 kHz, from which the slope is extracted. **c**, Illustration of a clock frequency adjustment step determined by the fitted error signal shape. In red we draw one exemplary data point with its corresponding error bar extracted from the measurement in panel b. The green arrow indicates the resulting clock adjustment step.

To operate the set-up as a clock, we first acquired a single absorption spectrum, as shown in Extended Data Fig. 2a. We observed that a Lorentzian line shape fits our absorption measurement data well. We then measured the expected error signal (Extended Data Fig. 2b). To measure an error signal, we modulated the offset lock frequency with 10 Hz between two frequencies *f*_low_ and *f*_high_. The difference Δ*f* = *f*_high_ − *f*_low_ was kept constant during the measurement, and only the centre was swept across the resonance. In our measurements, we used a frequency deviation Δ*f* of ${f}_{{\rm{FWHM}}}/\sqrt{3}$, with *f*_FWHM_ describing the FWHM of the measured absorption peak (Extended Data Fig. 2a). We fitted this signal with the function

$${y}_{{\rm{f}}{\rm{i}}{\rm{t}}}(f)=\frac{{A}_{{\rm{L}}}}{1+{\left(\frac{f+\Delta f/2}{\gamma }\right)}^{2}}-\frac{{A}_{{\rm{L}}}}{1+{\left(\frac{f-\Delta f/2}{\gamma }\right)}^{2}},$$

where *A*_L_ is the amplitude and *γ* is the half width at half maximum of the Lorentzian. For the clock operation, the laser frequency was again switched between two frequencies while we adjusted the centre position with the feedback. For both frequency positions, the counts *c*_low,*i*_ and *c*_high,*i*_ were recorded, where *i* is the measurement index for averaging the signal. The applied error signal *E* can be calculated as

$$E(c)={y}_{{\rm{fit}}}^{-1}\left(\frac{1}{L}\mathop{\sum }\limits_{i=1}^{L}\frac{{c}_{{\rm{high}},i}-{c}_{{\rm{low}},i}}{({c}_{{\rm{high}},i}+{c}_{{\rm{low}},i})/2}\right),$$

with the number of cycles *L* and the inverse fit function ${y}_{{\rm{fit}}}^{-1}(c)$. Extended Data Fig. 2c illustrates an adjustment step inferred by the calculation of *E*. With the integration times set in the clock operation, the beat was shifted after every interrogation cycle by the calculated value *E*, as shown in Extended Data Fig. 1.

### Variation of the fundamental constants

The variation of *α*(*t*) relates to the variation of the ratio of the ^229^Th and ytterbium clock frequencies through: $({k}_{\mathrm{Th}}^{\alpha }-{k}_{\mathrm{Yb}}^{\alpha })\times {\partial }_{t}\,\log \,\alpha (t)\,=$ ${\partial }_{t}\,\log ({f}_{\mathrm{Th}}/{f}_{\mathrm{Yb}})$. Here ${k}_{{\rm{Th}}}^{\alpha }$ and ${k}_{{\rm{Yb}}}^{\alpha }$ are sensitivity factors that describe how much the respective transition frequency depends on *α*. The log-derivative directly corresponds to the measured infrared beat frequency *f*_b_ through ${\partial }_{t}\log ({f}_{\mathrm{Th}}/{f}_{\mathrm{Yb}})=8({\partial }_{t}\,{f}_{{\rm{b}}})/{f}_{0}$, where the factor of 8 originates from the three frequency-doubling steps^64^.

The anomalously low energy of the ^229^Th nuclear transition arises from a coincidental near-cancellation of the mega-electronvolt-scale strong force and Coulomb contributions to the binding energies of the nuclear states involved^15^. We, therefore, expect that ${k}_{\mathrm{Th}}^{\alpha }\approx {k}_{\mathrm{Th}}^{{\rm{g}}}\approx {k}_{\mathrm{Th}}^{{m}_{q}}$, where ${k}_{{\rm{Th}}}^{g}$ and ${k}_{{\rm{Th}}}^{{m}_{q}}$ are the sensitivity constants that relate between the log-derivatives of the frequency ratio and *Λ*_QCD_ and *m*_*q*_, respectively. They by far dominate the sensitivity of the Yb^+^ reference for which the corresponding sensitivities are approximately 1 (ref. ^59^). We determined the amplitude of possible oscillations in the experimental data by computing the Lomb–Scargle power spectrum *P*_f_ of the beat signal translated into the VUV. It corresponds to an oscillation amplitude *A*_f_ through ${A}_{{\rm{f}}}=\sqrt{4{P}_{{\rm{f}}}/{N}_{\mathrm{tot}}}/{f}_{0}$, where *N*_tot_ is the number of data points^65^.

Various theories suggest that dark matter could consist of yet undiscovered ultralight scalar bosons^41^. Such bosons would interact only very weakly with other particles and their behaviour can be approximately described by a free field: $\phi =\frac{\sqrt{2{(\hbar c)}^{3}{\rho }_{\mathrm{DM}}}}{{m}_{\phi }{c}^{2}}\,\cos \left(\frac{{m}_{\phi }{c}^{2}}{\hbar }t+{\delta }\right)$. Here *ħ* is the reduced Planck constant, *ρ*_DM_ = 0.4 GeV cm^−^^3^ is the observed dark matter density in the Milky Way^41^, *m*_*ϕ*_ is the unknown mass of the boson and *δ* is some unknown phase. The coupling of such particles to matter can be expressed through the following Lagrangian density^42,56,66^:

$${\mathcal{L}}\subset -\kappa \phi \left[\frac{{d}_{{\rm{e}}}}{4{\mu }_{0}}{F}_{\mu \nu }{F}^{\mu \nu }-\frac{{d}_{{\rm{g}}}\,{\beta }_{3}}{2{g}_{3}}{G}_{\mu \nu }^{a}{G}^{a\mu \nu }+\sum _{q=u,d}({d}_{{m}_{q}}+{\gamma }_{{m}_{q}}{d}_{{\rm{g}}}){m}_{q}{c}^{2}{\bar{\psi }}_{q}{\psi }_{q}\right].$$

Here we included only the terms that are relevant for this study. *ϕ* is the scalar field, *F*_*μν*_ the electromagnetic field, ${G}_{\mu \nu }^{a}$ the gluon field and *ψ*_*q*_ the quark field. $\kappa =\frac{\sqrt{4{\rm{\pi }}}}{{M}_{\mathrm{Pl}}{c}^{2}}$, where *M*_Pl_ is the (unreduced) Planck mass. *μ*_0_ is the vacuum permeability and *β*_3_ is the QCD beta function that describes the running of the coupling constant *g*_3_. ${\gamma }_{{m}_{q}}{d}_{{\rm{g}}}$ describes the anomalous and *m*_*q*_ the bare contribution to the quark mass. The first term leads to a modification of the electromagnetic coupling strength *α* → *α* + *ακd*_e_*ϕ* (ref. ^66^). The amplitude of this oscillation is given by ${{\mathcal{A}}}_{{\rm{f}}}=\frac{\alpha \kappa {d}_{{\rm{e}}}\sqrt{2{(\hbar c)}^{3}{\rho }_{\mathrm{DM}}}}{{m}_{\phi }{c}^{2}}$. Finally, using the Lomb–Scargle power spectrum *P*_f_ of the VUV equivalent of the measured beat signal, we arrived at the following expression for the scalar-photon coupling strength:

$${d}_{{\rm{e}}}=\sqrt{\frac{{c}^{5}}{{\hbar }^{3}}}\frac{3{M}_{\mathrm{Pl}}}{\sqrt{8{\rm{\pi }}{\rho }_{\mathrm{DM}}}}\frac{1}{{k}_{\mathrm{Th}}^{\alpha }\,{f}_{0}}\sqrt{\frac{4{P}_{{\rm{f}}}}{{N}_{\mathrm{tot}}}}{m}_{\phi }.$$

Here we included a factor of 3 to account for possible stochastic fluctuations in the dark matter density^67^. The corresponding equations for *Λ*_QCD_ and *m*_*q*_ follow completely analogously and differ only by the respective sensitivity factor.

To evaluate the statistical significance of the amplitudes in the Lomb–Scargle power spectrum, we performed Monte Carlo simulations of the clock feedback loop. For this, we took *N* = *T* + 1 random values from the Poissonian distribution to simulate the actual experimental cycle. where *T* is the integration time in seconds. This distribution follows from the SNR of the Lorentzian fit of the measured data at positions ${f}_{\mathrm{est}}\pm \mathrm{FWHM}/2\sqrt{3}$. We then added white noise to these values with an amplitude that matched the noise we observed on the beat frequency. We then averaged these *N* values and used the error function to calculate a new estimate for *f*_est_, with which we repeated the procedure. We assumed a decrease in SNR for each subsequent random value to model the linear decrease in laser power that we experienced during the data taking. Extended Data Fig. 1a shows that the simulated beat frequency has the same characteristics as the measured one. We further saw that the power spectrum and Allan deviation of the simulated data fitted well to the experiment values.

For the experimental measurement acquired between 2 April 2026 (08:30:15 UTC) and 3 April 2026 (07:04:15 UTC), we extracted one beat-frequency value in each cycle to construct a sample of independent frequency ratios. The Lomb–Scargle periodograms for both the experimental data and simulations were computed using the Astropy Python package^68^ with the setting ‘normalization = psd’.

To assess the statistical significance of peaks in the experimental periodogram and account for the look-elsewhere effect, we estimated a 5% detection threshold. When finding a peak above this threshold, the probability of it being a false detection is less than *p*_0_ = 5%. Adopting the method of refs. ^52,65^, we performed 1,000 Monte Carlo simulations of the clock loop and computed the corresponding Lomb–Scargle periodograms. Then, we fitted the histogram of the cumulative power at each frequency with an exponential cumulative distribution function of the form: $1-\exp (-a({P}_{{\rm{f}}}-{P}_{0}))$. The fitting parameters, *a* and *P*_0_, allowed us to estimate the detection threshold *P*_f,th_ and the corresponding amplitude according to ${P}_{{\rm{f}},\mathrm{th}}={P}_{0}-\frac{1}{a}{\rm{ln}}[1-{(1-{p}_{0})}^{1/{n}_{\mathrm{ind}}}]$, where ${n}_{\mathrm{ind}}\approx {t}_{\mathrm{tot}}/T\approx$ 3,800 is the number of independent frequencies. Here the estimated threshold fits well to the analytical solution for an exponential distribution, which is expected for white noise. We found no amplitudes exceeding the detection threshold, indicating the absence of DM oscillations in our measurements.

The local 95% confidence level was determined using 1,000 Monte Carlo simulations. For each simulation, a random offset was added to the experimental beat frequency at each time step. The offset followed a Gaussian white noise distribution, with a standard deviation *σ* = 1.34 kHz, derived from the experimental beat frequency. The local 95% confidence level was then defined as the 95th percentile of the amplitude distribution at each frequency, allowing us to exclude, with 95% certainty, the presence of any oscillations with amplitudes exceeding this limit.

## Online content

Any methods, additional references, Nature Portfolio reporting summaries, source data, extended data, supplementary information, acknowledgements, peer review information; details of author contributions and competing interests; and statements of data and code availability are available at https://doi.org/10.1038/s41586-026-11084-4.

## Supplementary information

Peer Review File

## Data Availability

The data supporting the findings of this study are openly available in Zenodo at https://doi.org/10.5281/zenodo.22657608^69^.
