# Supplementary material for: A thorium-229 optical nuclear clock with feedback loop
Source: Nature. 2026 Oct 7;658(8137):897–902. doi: 10.1038/s41586-026-11084-4 (PMC13686927; doi:10.1038/s41586-026-11084-4)
Supplement: Supplementary file 1 [file 41586_2026_11084_MOESM1_ESM.pdf]

Peer Review File

A thorium-229 optical nuclear clock with feedback loop

Corresponding Author: Professor Thorsten Schumm

Any redactions in this file are there to maintain patient confidentiality, the confidentiality of unpublished data, or to remove third-party material.

This file contains all reviewer reports in order by version, followed by all author rebuttals in order by version.

Version 0:

Reviewer comments:

Referee #1

(Remarks to the Author)
The authors report the first realization of a nuclear clock in which the thorium-229 isomeric transition is used to directly stabilize a vacuum-ultraviolet (VUV) laser through a feedback loop. This represents a major advance over previous experiments, where the VUV lasers used to excite the thorium-229 nuclear transition were stabilized to external frequency references. Direct stabilization to the nuclear transition enables a substantial improvement in clock performance. By combining a recently developed continuous-wave 148 nm laser system with highly doped thorium-229 calcium fluoride crystals, the authors achieve a fractional frequency instability approaching 10^{-15} after one day of averaging.
The authors then use the nuclear clock to search for signatures of ultralight dark matter through measurements of oscillatory and slowly varying shifts in the nuclear transition frequency. Compared with previous thorium-229 experiments, the present system improves sensitivity by approximately one order of magnitude. Furthermore, the rapid frequency comparison enabled by the feedback loop extend the accessible mass range for ultralight dark matter searches. As no evidence for such time-dependent effects is observed, the authors derive new constraints on several couplings between ultralight dark matter and Standard Model fields. While the resulting bound on the photon coupling is comparable to those obtained with state-of-the-art optical atomic clocks based on electronic transitions, the constraints on couplings to gluons and light quarks are two to three orders of magnitude stronger. Remarkably, these limits surpass existing bounds from equivalence-principle tests over part of the accessible mass range.
Finally, the authors discuss several avenues for technical improvement, arguing that a stand-alone nuclear clock operating at the performance level of the best optical atomic clocks is achievable. Such an advance could improve sensitivity to ultralight dark matter by as much as four orders of magnitude.

Beyond the immediate technical achievement, this work represents a significant milestone for the emerging field of nuclear clocks. The demonstration of direct laser stabilization to the thorium-229 nuclear transition establishes a new experimental paradigm and provides the first convincing evidence that a fully operational nuclear frequency standard is within reach. The results are both novel and impactful, combining advances in VUV laser technology, crystal-based thorium spectroscopy, and precision frequency metrology. The experimental methodology is sound and carefully validated, with systematic effects and statistical uncertainties thoroughly characterized and appropriately propagated to the final results. The dark-matter analysis is performed using well-established techniques and relies on a robust frequency dataset acquired over extended periods of operation. The manuscript is clearly written, logically organized, and accessible to a broad readership. In particular, the authors provide an excellent overview of the scientific motivation, placing the work in the context of both next-generation frequency standards and searches for physics beyond the Standard Model. Overall, the study constitutes a substantial advance in precision measurement science and will likely stimulate further developments in nuclear clocks and their applications to fundamental physics.

In view of the significance of the results for precision metrology and fundamental physics, and the high quality of the experimental work, I strongly recommend publication of this manuscript in Nature.

Referee #2

(Remarks to the Author)
This manuscript reports the first realization of an operational optical nuclear clock based on the low-energy isomeric

transition in 229Th. The authors demonstrate a closed-loop frequency stabilization scheme where the absorption spectrum of the nuclear transition is utilized to obtain the error signal for the feedback loop to their 148 nm laser. The absolute frequency of the laser is calibrated by a fiber link to an Yb+ ion clock at BEV. They achieved a fractional instability of 3*10^-12/sqrt(tau) and run-to-run fluctuation of 5*10^-13. With this data, they demonstrated a constraint on the existence of scalar ultralight dark matter, which already showed substantially better performance compared to conventional microwave or optical clocks.

The nuclear clock is a recent hot topic, a resonant frequency for which is narrowed down to ~1 kHz level in 2024 for the first time. Multiple groups in the world are working on making a clock with this transition, and to the best knowledge of the author, this manuscript is the first report on realization of locking a clock laser to the nuclear transition. In spite of the limited signal, by using an absorption spectroscopy, authors effectively obtained a signal for the feedback loop, and implemented the feedback. The methodology and the demonstration of the result follows a standard procedure in the atomic clock community, presenting the current achievement and future direction clearly. Particular strength of this manuscript is that it showed the extensibility of the system in the future by showing the currently achievable constraint in the ultralight dark matter search.

Overall, the manuscript shows a clear achievement of a milestone for the nuclear clock with a scientific benefit of pursuing better stability. Before the manuscript can be published, following points should be clarified.
The introduction is somewhat lengthy. Can at least a part of it be substituted by citing recent review papers, e.g., Appl. Phys. Rev. 12, 041331 (2025) and arXiv:2606.11048 (2026). Particularly, the first paragraph of p.3 is essentially a history of the accuracy improvement in the clock transition, and Table VI of the first review paper is a good summary of this paragraph.
At the end of page 7, authors states “precise values of these sensitivity factors can currently not be calculated [15,42].” Ref. 42 is slightly older publication with prediction K=10^4-10^5 and Ref. 15 is newer, with K=5900(2300). Ref. 15 cites Ref. 42 implying that Ref. 15 took Ref. 42 into account and obtained more precise number. However, the authors of this manuscript only used number on Ref. 15 to obtain the plot in Fig. 4. It would be more respectful to authors of Ref. 15 to do one of the following.
Describe justification on why authors think numbers in Ref. 42 is more reliable than that in Ref. 15.
make use of number in Ref. 15 to generate plots in Fig. 4. This can be either adding another line for K=5900 or replace lines for K=10^4 and 10^5 with K=5900.
In Fig. 6, the overall width for beat frequency deviation looks gradually increases as time increases. On the other hand, in Fig. 2 does not have large average time to show this effect that is visible at the time scale >40000 s. Can the Allan deviation plot extended to a bit longer average time?
There are some typos.
p.3, line 4: 7.8(5) eV [19,20] -> 7.6(5) eV, as far as the reviewer checked Ref. 19
Fig. 4.b: the label k^alpha_Th seems opposite, i.e., correct one should be 10^4 for the upper line and 10^5 for the lower line.
Page number 9147 is missing in Ref. [15].

Referee #3

(Remarks to the Author)
The manuscript "A thorium-229 optical nuclear clock with feedback loop" by L. Toscani De Col et al. presents an experimental demonstration of stabilization of a CW laser frequency to the thorium-229 148-nm nuclear transition. This is the first demonstration in which the nuclear clock has been operated as a fully functional clock. The absorption spectroscopy of the nuclear transition of thorium-229 embedded in a room-temperature CaF2 crystal enables rapid feedback on the laser frequency on a timescale shorter than the isomer lifetime. The authors compared their nuclear clock with the Yb+ E3 frequency standard and reported constraints on models of ultralight dark matter. The frequency reproducibility and its dependence on the position of the host crystal are also experimentally investigated, which will have significant importance for the future improvement of solid-state nuclear clocks.

I found the paper to be well written and sound in its physics. Given its significant impact, this work is worth publishing in Nature. However, I have a major concern about one of the authors’ statements regarding the constraints on models of ultralight dark matter (UDM).

I do not recommend publication of the manuscript in the current form. However, if I have misunderstood any essential details, I welcome a rebuttal from the authors.

Here are my concerns and suggestions for improvement:

1.I question the validity of the following statements in the abstract:"We use the nuclear clock to constrain models of ultralight dark matter” and “these constraints compete with the best atomic clocks concerning dark matter coupling to photons and go beyond previous measurements regarding coupling to the strong force and quarks.” The basis of these claims is summarized in Figure 4, where the authors assume k_Th = 10^4 and k_Th = 10^5 to draw their conclusions without sufficient justification. The authors state on page 7 that “precise values of these sensitivity factors can currently not be calculated [15, 42], but it is predicted that they will exceed those of the most sensitive atomic clock transitions by multiple orders of magnitude.” If these sensitivity factors cannot be calculated precisely and are only predicted to be larger than those of existing atomic clocks, how can the authors justify assuming such large sensitivity factors (e.g., 10^4 or 10^5) to claim that they have constrained UDM models and gone beyond previous measurements? Furthermore, why was a more conservative value, such as k_Th = 10^3, not considered? Is there any physical justification for ruling out k_Th = 10^3? I strongly recommend that the authors carefully distinguish between their actual achievements and their underlying assumptions.

2.I recommend including “R. Elwell et al., Nature 648, 300 (2025)” in the sentence “Thereafter, resonant laser excitation…” (page 3) because it is also a milestone achievement in this context.

3.In the second paragraph of the “Clock operation” section, the authors state “SNR = 17”. Did the authors derive this SNR value from a fit of the Allan deviation data or from other independent spectroscopic measurements? Such information would help readers understand how the authors plot the “Shot noise limit” line in Figure 2.

4.In the same paragraph, it may be better to replace “the absorption amplitude A” with “the fractional absorption amplitude A” to describe the parameter more appropriately.

5.In the first paragraph of the “Clock performance” section, the authors state “it rises for both measurements as sqrt(tau).” What do the authors imply here? Is the sqrt(tau) dependence important? It depends on the clock laser’s intrinsic instability and feedback gain. For example, if the intrinsic instability of the clock laser at 1 s is 5x10^-14, the line would become flat (not following sqrt(tau)) for the operation mode of “T = 30 min” until the averaging time reaches 30 min.

6.In Figure 2, why are the errors for the last two data points (around the averaging time of 10^4 s) smaller than those of the data points at 2000 s and 4000 s? The overlapping Allan deviation errors typically increase with longer averaging times, as the number of (independent) data points decreases.

7.In Figure 3b, could the authors specify the diameter of the 148-nm laser at the crystal? Here, the authors discuss displacements of the beam position by 0.5-1 mm. Is 0.5-1 mm larger or smaller than the diameter of the spectroscopy laser?

8.In the caption of Figure 3, “T” and “t_tot” should be in italics (“tot” should not be in italics).

9.On page 6, “to perform measurements on five crystal positions”. Should this be “four crystal positions” since scans #2 and #4 in Fig. 3b used the same crystal position?

10.In Figure 4b, should k_Th^alpha = 10^5 and k_Th^alpha = 10^4 be reversed?

11.In Figures 4b-d, I recommend replacing “229Th/Yb” with “229Th/Yb+ E3” to avoid misunderstanding between the Yb+ E3 clock and the neutral ytterbium optical lattice clock. The “BEV:Yb+ clock” in Figure 1 should also be replaced with “BEV:Yb+ E3 clock” to clarify that this Yb+ clock is based on the E3 transition rather than the E2 transition.

12.Could the authors add an explanation of what the red data point, its error bar, and the green arrow in Figures 5b and 5c represent?

13.Figure 6 caption: “blue” -> “black”

14.On page 16, “poissonian” -> “Poissonian” (“P” should be capitalized.)

Version 1:

Reviewer comments:

Referee #2

(Remarks to the Author)
The authors provided quite reasonable responses and modifications to the points reviewers mentioned. The manuscript now looks ready to be published as the current status.

Referee #3

(Remarks to the Author)
I have reviewed the second version of the manuscript. I found my questions and comments have been exhaustively discussed. I am therefore now in favor of publication of the manuscript in Nature.

One final comment: in their response regarding the sqrt(tau) dependence in the stability plot, the authors state “We have therefore shortened the description to focus the attention on the latter”. I may have overlooked this in the revised manuscript, but could the authors please clarify which specific parts of the descriptions have been shortened?

Open Access This Peer Review File is licensed under a Creative Commons Attribution 4.0 International License, which permits use, sharing, adaptation, distribution and reproduction in any medium or format, as long as you give appropriate credit to the original author(s) and the source, provide a link to the Creative Commons license, and indicate if changes were made.
In cases where reviewers are anonymous, credit should be given to 'Anonymous Referee' and the source.
The images or other third party material in this Peer Review File are included in the article's Creative Commons license, unless indicated otherwise in a credit line to the material. If material is not included in the article's Creative Commons license and your intended use is not permitted by statutory regulation or exceeds the permitted use, you will need to obtain permission directly from the copyright holder.
To view a copy of this license, visit https://creativecommons.org/licenses/by/4.0/

Dear Editor,

We thank all three reviewers for the invested time and very valuable and constructive feedback. We have revised the manuscript to address all comments and suggestions. Below, we provide a point-by-point response (reviewers' comments in red, our replies and manuscript changes in blue). We also provide a resubmitted version of the article in a separate document, where all changes made to the manuscript are clearly highlighted.

On behalf of all authors,

Ekkehard Peik, Thorsten Schumm

Reviewer #1 (Remarks to the Author):

The authors report the first realization of a nuclear clock in which the thorium-229 isomeric transition is used to directly stabilize a vacuum-ultraviolet (VUV) laser through a feedback loop. This represents a major advance over previous experiments, where the VUV lasers used to excite the thorium-229 nuclear transition were stabilized to external frequency references. Direct stabilization to the nuclear transition enables a substantial improvement in clock performance. By combining a recently developed continuous-wave 148 nm laser system with highly doped thorium-229 calcium fluoride crystals, the authors achieve a fractional frequency instability approaching 10-15 after one day of averaging. The authors then use the nuclear clock to search for signatures of ultralight dark matter through measurements of oscillatory and slowly varying shifts in the nuclear transition frequency. Compared with previous thorium-229 experiments, the present system improves sensitivity by approximately one order of magnitude. Furthermore, the rapid frequency comparison enabled by the feedback loop extend the accessible mass range for ultralight dark matter searches. As no evidence for such time-dependent effects is observed, the authors derive new constraints on several couplings between ultralight dark matter and Standard Model fields. While the resulting bound on the photon coupling is comparable to those obtained with state-of-the-art optical atomic clocks based on electronic transitions, the constraints on couplings to gluons and light quarks are two to three orders of magnitude stronger. Remarkably, these limits surpass existing bounds from equivalence-principle tests over part of the accessible mass range. Finally, the authors discuss several avenues for technical improvement, arguing that a stand-alone nuclear clock operating at the performance level of the best optical atomic clocks is achievable. Such an advance could improve sensitivity to ultralight dark matter by as much as four orders of magnitude. Beyond the immediate technical achievement, this work represents a significant milestone for the emerging field of nuclear clocks. The demonstration of direct laser stabilization to the thorium-229 nuclear transition establishes a new experimental paradigm and provides the first convincing evidence that a fully operational nuclear frequency standard is within reach. The results are both novel and impactful, combining advances in VUV laser technology, crystal-based thorium spectroscopy, and precision frequency metrology. The experimental methodology is sound and carefully validated, with systematic effects and statistical uncertainties thoroughly characterized and appropriately propagated to the final results. The dark-matter analysis is performed using well-established techniques and relies on a robust frequency dataset acquired over extended periods of operation. The manuscript is clearly written, logically organized, and accessible to a broad readership. In particular, the authors provide an excellent overview of the scientific motivation, placing the work in the context of both next-generation frequency standards and searches for physics beyond the Standard Model. Overall, the study constitutes a substantial advance in precision measurement science and will likely stimulate further developments in nuclear clocks and their applications to fundamental physics. In view of the significance of the results for precision metrology and fundamental physics, and

the high quality of the experimental work, I strongly recommend publication of this manuscript in Nature.

We thank the Reviewer 1 for the positive assessment of our work.

Reviewer #2 (Remarks to the Author):

This manuscript reports the first realization of an operational optical nuclear clock based on the low-energy isomeric transition in 229Th. The authors demonstrate a closed-loop frequency stabilization scheme where the absorption spectrum of the nuclear transition is utilized to obtain the error signal for the feedback loop to their 148 nm laser. The absolute frequency of the laser is calibrated by a fiber link to an Yb+ ion clock at BEV. They achieved a fractional instability of 3*10-12/sqrt(tau) and run-to-run fluctuation of 5*10-13. With this data, they demonstrated a constraint on the existence of scalar ultralight dark matter, which already showed substantially better performance compared to conventional microwave or optical clocks. The nuclear clock is a recent hot topic, a resonant frequency for which is narrowed down to 1 kHz level in 2024 for the first time. Multiple groups in the world are working on making a clock with this transition, and to the best knowledge of the author, this manuscript is the first report on realization of locking a clock laser to the nuclear transition. In spite of the limited signal, by using an absorption spectroscopy, authors effectively obtained a signal for the feedback loop, and implemented the feedback. The methodology and the demonstration of the result follows a standard procedure in the atomic clock community, presenting the current achievement and future direction clearly. Particular strength of this manuscript is that it showed the extensibility of the system in the future by showing the currently achievable constraint in the ultralight dark matter search. Overall, the manuscript shows a clear achievement of a milestone for the nuclear clock with a scientific benefit of pursuing better stability. Before the manuscript can be published, following points should be clarified.

introduction The introduction is somewhat lengthy. Can at least a part of it be substituted by citing recent review papers, e.g., Appl. Phys. Rev. 12, 041331 (2025) and arXiv:2606.11048 (2026). Particularly, the first paragraph of p.3 is essentially a history of the accuracy improvement in the clock transition, and Table VI of the first review paper is a good summary of this paragraph.

We agree with the reviewer that the introduction could be shortened and have streamlined it by deleting some phrases. Still, in view of the very broad readership of Nature and the aspect of self-containedness, we find it important to present a concise overview of the evolution of the nuclear transition energy from 100 eV uncertainty down to pico electron Volts.

We also agree to include review articles for readers interested in more details and the mentioned table indeed provides a comprehensive overview. We have added the suggested Appl. Phys. Rev. 12, 041331 (2025), but would refrain from including arXiv:2606.11048 (2026), as it is unpublished work in progress (submitted after this manuscript). We now cite the established Nature Reviews Physics 3, 238–248 (2021).

At the end of page 7 authors states "precise values of these sensitivity factors can currently not be calculated [15, 42]." Ref. 42 is slightly older publication with prediction $K = 10^4 - 10^5$ and Ref. 15 is newer, with K=5900(2300). Ref. 15 cites Ref. 42 implying that Ref. 15 took Ref. 42 into account and obtained more precise number. However, the authors of this manuscript only used number on Ref. 15 to obtain the plot in Fig. 4. It would be more respectful to authors of Ref. 15 to do one of the following. Describe justification on why authors think numbers in Ref. 42 is more reliable than that in Ref. 15. make use of number in Ref. 15 to generate plots in Fig. 4. This can be either adding another line for K=5900 or replace lines for $K = 10^4$ and 10^5 with K=5900.

First, we would like to point out that we mean no disrespect to the authors of Ref. [15] (Beeks et al.). Jun Ye's team at JILA and Marianna Safronova from Delaware are among our closest collaborators; the experimental results that form the basis of this work were obtained using crystals from Vienna. A good fraction of the authors of Ref. [15] from the Vienna team is also among the authors of the current manuscript.

The assessments made in Ref. [42] (Caputo et al.) are still highly relevant. There, it was shown that the enhancement factor k_{Th} strongly varies, depending on the different nuclear parameters. It was shown that enhancement factors k_{Th}=10^3-10^5 are to be expected. Smaller enhancement factors are possible but quite unlikely, as they require very specific cancellation for which there is currently no physical basis.
The value of $k_{\mathrm{Th}} = 5900(2300)$ in Ref. [15] (Beeks et al.) is specifically given for the semi-classical prolate-spheroid model without octupole deformation. (2300) describes the uncertainty that follows from measurement uncertainties of nuclear parameters. However, it does not take into account the theoretical uncertainty of the used nuclear model. It could be significantly larger. In the submission version of the manuscript, we had chosen $k_{\mathrm{Th}} = 10^4$ as the lower estimate of the enhancement factor, following the choice made in Ref. [47] (Arakawa et al.), also to enable a direct comparison of these works, addressing complementary regimes and timescales. Note that also this work includes authors from Ref. [15] and [42].

We understand the concern of the reviewer (also in view of a similar comment by Reviewer #3) of not representing the limits that would be achieved with even lower enhancement factors. We therefore added a lower band indicating an even more conservative $k_{\mathrm{Th}} = 10^3$.

In Fig. 6 the overall width for beat frequency deviation looks gradually increases as time increases. On the other hand, in Fig. 2 does not have large average time to show this effect that is visible at the time scale > 40000 s. Can

the Allan deviation plot extended to a bit longer average time?

The observation that the instability of the beat frequency increases over time is correct. This is due to the decreasing laser power. It started at 70 pW and decreased to 30 pW, which means the SNR reduced from 20 to 13, in the 23 h clock operation. Because the Allan deviation for each averaging time τ is calculated from all data points, the gradually increasing frequency noise does not only affect long averaging times but all timescales, under the reasonable assumption that the spectrum of shot noise remains white. All the data shown in Fig. 6a contributes to the calculated Allan deviation and is represented in the Allan deviation plot (Fig. 2).

Extending the calculation of the Allan deviation to longer averaging times would lead to data points of higher statistical uncertainty. We therefore adhere to the widely applied convention of plotting the Allan deviation for averaging times up to about one-quarter of the total measurement duration only.

In response to a comment of Reviewer #3, we have modified the error bars in Fig. 2., see also the explanations below.

Typos There are some typos. p.3, line 4: 7.8(5) eV [19,20] $->$ 7.6(5) eV, as far as the reviewer checked Ref. 19 Fig. 4.b: the label k_{Th}^{alpha} seems opposite, i.e., correct one should be 10^4 for the upper line and 10^5 for the lower line. Page number 9147 is missing in Ref. [15].

The use of the value 7.8(5) eV in connection with Refs. [19, 20] is intentional. The 2007 PRL by Beck et al. indeed proposed a value of 7.6(5) eV. The same team later re-analyzed their data in 2009, taking into account a previously ignored iter-band transition (2 % branching ratio) from the 42.43 keV 7/2+ [633] state to the isomeric 3/2+ [631] state. The community predominantly adopted this slightly higher value.

Fig 4 b) is corrected.

The page number is corrected in Ref. [15].

Reviewer #3 (Remarks to the Author):

The manuscript "A thorium-229 optical nuclear clock with feedback loop" by L. Toscani De Col et al. presents an experimental demonstration of stabilization of a CW laser frequency to the thorium-229 148-nm nuclear transition. This is the first demonstration in which the nuclear clock has been operated as a fully functional clock. The absorption spectroscopy of the nuclear transition of thorium-229 embedded in a room-temperature CaF2 crystal enables rapid feedback on the laser frequency on a timescale shorter than the isomer lifetime. The authors compared their nuclear clock with the Yb+ E3 frequency standard and reported constraints on models of ultralight dark matter. The frequency reproducibility and its dependence on the position of the host crystal are also experimentally investigated, which will have significant importance for the future improvement of solid-state nuclear clocks. I found the paper to be well written and sound in its physics. Given its significant impact, this work is worth publishing in Nature. However, I have a major concern about one of the authors' statements regarding the constraints on models of ultralight dark matter (UDM). I do not recommend publication of the manuscript in the current form. However, if I have misunderstood any essential details, I welcome a rebuttal from the authors. Here are my concerns and suggestions for improvement:

abstract I question the validity of the following statements in the abstract:"We use the nuclear clock to constrain models of ultralight dark matter" and "these constraints compete with the best atomic clocks concerning dark matter coupling to photons and go beyond previous measurements regarding coupling to the strong force and quarks." The basis of these claims is summarized in Figure 4, where the authors assume $k_{\mathrm{Th}} = 10^4$ and $k_{\mathrm{Th}} = 10^5$ to draw their conclusions without sufficient justification. The authors state on page 7 that "precise values of these sensitivity factors can currently not be calculated [15, 42], but it is predicted that they will exceed those of the most sensitive atomic clock transitions by multiple orders of magnitude." If these sensitivity factors cannot be calculated precisely and are only predicted to be larger than those of existing atomic clocks, how can the authors justify assuming such large sensitivity factors (e.g., 10^4 or 10^5) to claim that they have constrained UDM models and gone beyond previous measurements? Furthermore, why was a more conservative value, such as $k_{\mathrm{Th}} = 10^3$, not considered? Is there any physical justification for ruling out $k_{\mathrm{Th}} = 10^3$? I strongly recommend that the authors carefully distinguish between their actual achievements and their underlying assumptions.

In Ref. [42] (Caputo et al.) and Ref. [15] (Beeks et al.) it is shown how the enhancement factor could vary with experimentally measured nuclear parameters that need to be measured at higher precision. The choice of model can also affect this value, and requires further theoretical study. However, it is shown that large enhancement factors are more plausible and a very small factor can only arise in certain accidental cancellations of high values, for which there is no physical basis. In [47] (Arakawa, et al.), which was written by authors from [42]

and [15], the same values of $k_{\mathrm{Th}} = 10^4$ and $k_{\mathrm{Th}} = 10^5$ were chosen to indicate the results with certain enhancement factors. We chose to use these values to allow direct comparisons between the manuscripts.

We understand the concern of Reviewers #2 and #3 of not representing the limits that would be achieved with even lower enhancement factors. Therefore we now also include a third band for $k_{\mathrm{Th}} = 10^3$.

We continue to support the claim: "these constraints compete with the best atomic clocks concerning dark matter coupling to photons and go beyond previous measurements regarding coupling to the strong force and quarks." Even in the quite unlikely case of a k_{Th} in the 10^2 range, the limit derived from the Th-229 clock measurement would still surpass all previous atomic clock measurements regarding m_q and Λ_{QCD}.

page 3 I recommend including "R. Elwell et al., Nature 648, 300 (2025)" in the sentence "Thereafter, resonant laser excitation…" (page 3) because it is also a milestone achievement in this context.

We have included the reference and slightly adapted the wording to accomodate it.

In the second paragraph of the "Clock operation" section the authors state "SNR = 17". Did the authors derive this SNR value from a fit of the Allan deviation data or from other independent spectroscopic measurements? Such information would help readers understand how the authors plot the "Shot noise limit" line in Figure 2.

The "SNR = 17" is calculated from independent measurements given in the same paragraph. The seperately measured Allan deviation follows the shot noise curve that is derived from this SNR value. This shows that it is indeed shot-noise-limited. We modified the wording in the related paragraph and the caption of Fig. 2 to make this point more clear.

In the same paragraph it may be better to replace "the absorption amplitude A" with "the fractional absorption amplitude A" to describe the parameter more appropriately.

We modified the expression as suggested.

In the first paragraph of the "Clock performance" section the authors state "it rises for both measurements as sqrt(tau)." What do the authors imply here? Is the sqrt(tau) dependence important? It depends on the clock laser's intrinsic instability and feedback gain. For example, if the intrinsic instability of the

clock laser at 1 s is $5x10^-14$, the line would become flat (not following sqrt(tau)) for the operation mode of "T = 30 min" until the averaging time reaches 30 min.

We had included this sentence to describe the trend of the fractional frequency instability for averaging times $\tau < T$, depicted in the figure. We fully agree with the referee that the rising slope depends on parameters of the servo system, as briefly discussed in the section 'Clock operation'. For averaging times $\tau > T$, the instability is determined by the shot noise limited interrogation of the Th-229 nuclear transition, which is the relevant observation here. We have therefore shortened the description to focus the attention on the latter.

In Figure 2 why are the errors for the last two data points (around the averaging time of 10^4 s) smaller than those of the data points at 2000 s and 4000 s? The overlapping Allan deviation errors typically increase with longer averaging times, as the number of (independent) data points decreases.

We thank the referee for raising this point. The Allan deviation errors were calculated as double-sided 95.5% confidence intervals using the χ^2 distribution, where the underlying equivalent number of χ^2 degrees of freedom is automatically adapted to the noise type which dominates at the respective averaging times. This approach is the default setting of the Stable32 program, a standard tool within the metrology community.

We agree with the reviewer that this is likely not the best model for evaluating the uncertainties in our system. We now modified the error bars to instead show the 68.3% confidence intervals assuming fixed degrees of freedom of white frequency noise. This model is to be preferred as it gives a adequate description of our system while being simpler. Now the error bars for the last data points do indeed increase.

In Figure 3b could the authors specify the diameter of the 148-nm laser at the crystal? Here, the authors discuss displacements of the beam position by 0.5-1 mm. Is 0.5-1 mm larger or smaller than the diameter of the spectroscopy laser?

We thank the referee for pointing out this omission; our beam size at the crystal is less than 0.5 mm. We verify this by guiding the beam through a 0.5 mm pinhole at the crystal position. We have added this information to the main text.

In the caption of Figure 3 "T" and "t_{tot}" should be in italics ("tot" should not be in italics).

We corrected the mistake.

On page 6 "to perform measurements on five crystal positions". Should this be "four crystal positions" since scans nr.2 and nr.4 in Fig. 3b used the same

crystal position?

We now corrected this.

In Figure 4b should $k_{Th}^{alpha} = 10^5$ and $k_{Th}^{alpha} = 10^4$ be reversed?

Thank you for spotting this mistake. It has now been corrected. We further removed the curve for "Si/H" as it was redundant.

In Figures 4b-d I recommend replacing "229Th/Yb" with "229Th/Yb+ E3" to avoid misunderstanding between the Yb+ E3 clock and the neutral ytterbium optical lattice clock. The "BEV:Yb+ clock" in Figure 1 should also be replaced with "BEV:Yb+ E3 clock" to clarify that this Yb+ clock is based on the E3 transition rather than the E2 transition.

Thank you for the comment. We added the "+" now to clarify that we compare to an ion clock. To avoid cluttered text in Fig. 4, we decided to only note the specific clock transitions for the Yb^+E2/E3 comparison. We added the missing information on the Yb^+ clock transition that we used in the methods now.

fig 5b and 5c Could the authors add an explanation of what the red data point, its error bar, and the green arrow in Figures 5b and 5c represent?

We now added a remark in the figure caption to explain the red data point with error bar as well as the green arrow.

Figure 6 caption: "blue" $->$ "black"

We corrected the mistake.

On page 16 "poissonian" $->$ "Poissonian" ("P" should be capitalized.)

We corrected the mistake.

Dear Editor,

We thank the reviewers for once again investing the time to check our manuscript. Below, we provide a response (reviewers' comments in red, our replies and manuscript changes in blue) to the last open query of referee 3. We have also shortened the abstract to comply with the word limit.

On behalf of all authors,

Ekkehard Peik, Thorsten Schumm

Reviewer #3:

One final comment: in their response regarding the sqrt(tau) dependence in the stability plot, the authors state “We have therefore shortened the description to focus the attention on the latter”. I may have overlooked this in the revised manuscript, but could the authors please clarify which specific parts of the descriptions have been shortened?

We apologize for an omission and thank the referee for spotting it: this change as described in our response letter had not been implemented in the manuscript. We have done this now.

In the section ”clock performance”, we had the statement:

In Fig. 2 we show the overlapping Allan deviation σ_y [39] plotted versus the averaging time τ. Until averaging times of $\tau \approx T$, it rises for both measurements as $\propto \sqrt{\tau}$. After this, the instability becomes mostly independent of the cavity and is instead determined by the shot noise-limited...

This has now been replaced by:

In Fig. 2 we show the overlapping Allan deviation σ_y [39] plotted versus the averaging time τ. For $\tau > T$, the instability is determined by the shot noise-limited...
